# Hesperidin inhibits colon cancer progression by downregulating SLC5A1 to suppress EGFR phosphorylation

**DOI:** 10.7150/jca.104867

**Published:** 2025-01-01

**Authors:** Xiaodong Li, Zhao Wu, Lebin Yuan, Xing Chen, He Huang, Fei Cheng, Wei Shen

**Affiliations:** 1 Department of Gastrointestinal Surgery, The Second Affiliated Hospital, Jiangxi Medical College, Nanchang University, China.; 2 Department of General Surgery, The Second Affiliated Hospital, Jiangxi Medical College, Nanchang University, China.

**Keywords:** hesperidin, colon cancer, SLC5A1, EGFR

## Abstract

**Objective:** Hesperidin, an active constituent of traditional Chinese medicine, Chenpi, exhibits anticancer properties across different cancers. This study aimed to clarify the efficacy of Hesperidin against tumors and its mechanisms of action in colon cancer.

**Method**: We assessed the efficacy of Hesperidin on human colon cancer cells (HCT-116 and DLD-1) and normal colonic epithelial cells (NCM460). We quantified cell viability at various Hesperidin concentrations using the CCK8 assay in a series of experiments. We employed clone formation, EdU incorporation, Transwell, and wound healing assays to clarify Hesperidin efficacy on cancer cell proliferation, invasion, and migration. Western blot analyses revealed modulations in epithelial-mesenchymal transition-related proteins, SLC5A1, EGFR, and phosphorylated EGFR levels following Hesperidin exposure. Co-IP assays further validated the interaction between SLC5A1 and EGFR. Our findings were significantly restored following SLC5A1 overexpression in colon cancer cells, highlighting its pivotal role in Hesperidin-induced responses.

**Results**: Hesperidin selectively impaired the viability of HCT-116 and DLD-1 colon cancer cells at specific concentrations while preserving normal NCM460 cells. This flavonoid dose-dependently reduced cancer cell proliferation, migration, and invasion. It significantly suppressed SLC5A1 and phosphorylated EGFR expression. We identified a direct SLC5A1-EGFR interaction essential for regulating EGFR activity in colon cancer. Overexpressing SLC5A1 significantly reduced the inhibitory effects of Hesperidin, highlighting its crucial role in this context.

**Conclusion:** Hesperidin exerts its anticancer effects on colon cancer by inhibiting SLC5A1 expression and consequently downregulating EGFR phosphorylation.

## Introduction

Colon cancer is among the most prevalent gastrointestinal cancers globally, with a recent increase in incidence among individuals under 50 years old 1, 2. Surgery is the first treatment step, frequently followed by adjuvant chemoradiotherapy. However, the 5-year survival rate for advanced colon cancer is 12% 3. It is essential to develop new treatments or methods to increase the chances of survival for patients with colon cancer.

**Figure 1:**
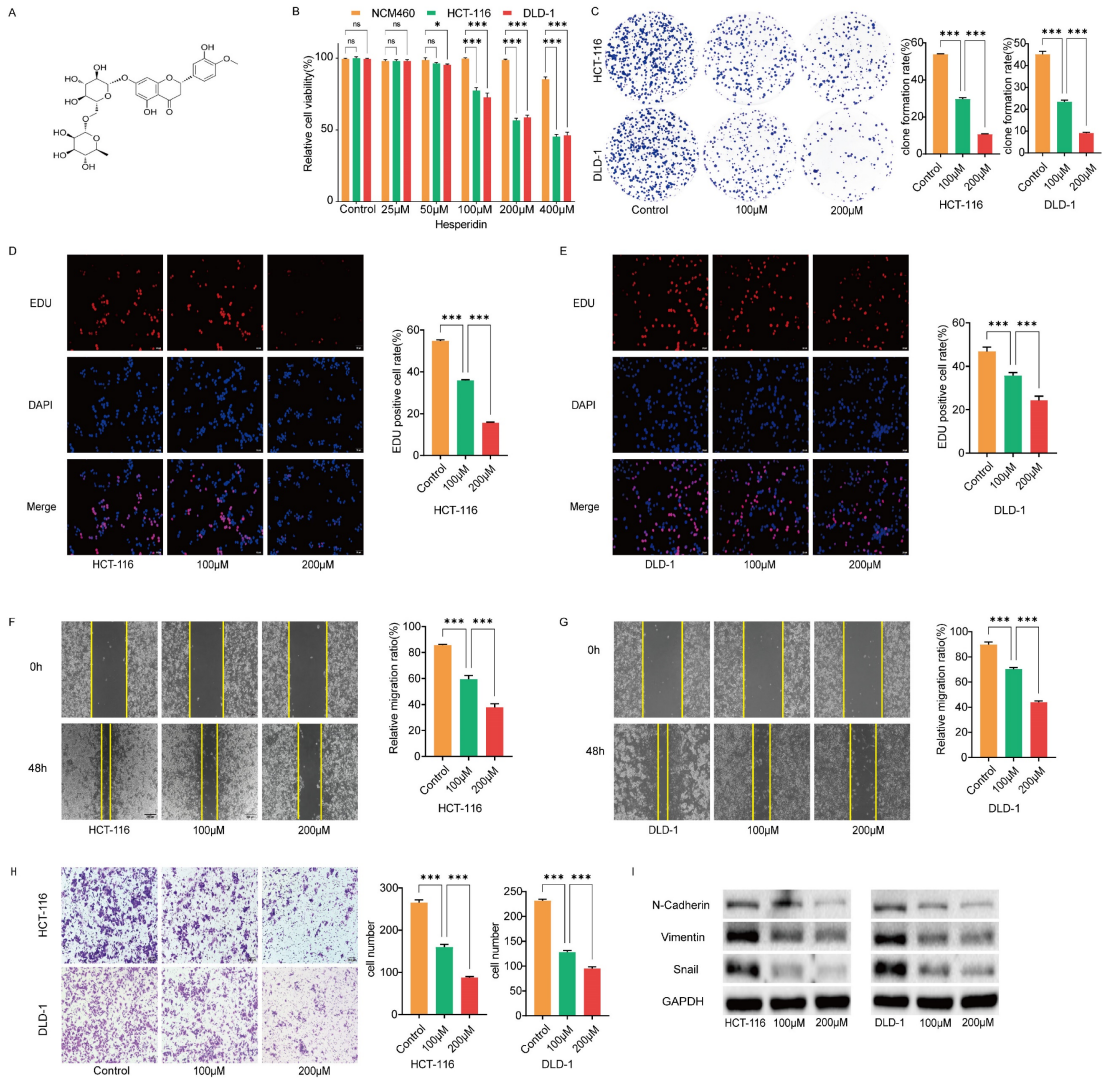
** Hesperidin inhibits proliferation, migration and invasion of colon cancer cells. (A)** The chemical structure of Hesperidin.** (B)** Cell viability was assayed by CCK-8 assay after treatment of NCM460, HCT-116 and DLD-1 cells with 0μM, 25μM, 50μM, 100μM, 200μM and 400μM Hesperidin for 48 hours. Colony formation assay **(C)** and EdU assay **(D-E)** were conducted to detect the effects of 0μM, 100μM and 200μM Hesperidin on the proliferative ability of HCT-116 and DLD-1 cells. **(F-G)** Wound healing assay was conducted to detect the effects of Hesperidin on the migration ability of HCT-116 and DLD-1 cells.** (H)** Transwell assay was conducted to detect the effects of Hesperidin on the invasion ability of HCT-116 and DLD-1 cells. **(I)** The expression of the EMT-related proteins was detected by Western boltting. All experiments were repeated three times and the data were represented as mean±SD. *p<0.05; **p<0.01; ***p<0.001; ns, *p*>0.05.

Hesperidin (dihydroflavonoid glycosides) is found in lemons, oranges, and other plants and is an essential component of the traditional Chinese medicine, Chenpi 4, 5. Figure 1A depicts the chemical structure of Hesperidin. Extensive investigations confirm its potent antitumoral properties across different cancers, including prostate 6, breast 7, cervical 8 and lung cancers 9. While these findings highlight the potent anticancer potential of Hesperidin, its specific effect on colon cancer is unclear.

The solute carrier family 5 (sodium/glucose cotransporter), member 1 (SLC5A1), also known as SGLT1, encodes an active glucose transporter that belongs to the sodium-dependent glucose transporter (SGLT) family. Increasing evidence demonstrates the involvement of SGLT in cancer-related biological processes, highlighting their regulatory role in key signaling pathways in carcinogenesis 10. A previous study reported that SGLT2 is highly expressed in breast cancer, and SGLT2 inhibitors can inhibit cell cycle arrest and apoptosis through the AMPK pathway 11. Additionally, SGLT2 inhibitors exhibit anticancer properties in liver cancer and glioblastoma 12, 13. SLC5A1 is upregulated in colorectal cancer and associated with an advanced clinical stage of the disease 14, indicating a potential role for SLC5A1 in colon cancer. However, the specific molecular mechanism of its action in colon cancer is unclear.

The epidermal growth factor receptor (EGFR) coordinates signaling pathways that regulate cell survival, migration, and growth, and these pathways are essential for various cellular functions 15. EGFR is overexpressed in many solid tumors, including breast, pancreatic, head and neck, prostate, ovarian, renal, colon, and non-small cell lung cancers 16. As a result, this study aimed to investigate whether Hesperidin could modulate EGFR phosphorylation through SLC5A1 downregulation to mediate the proliferation, invasion, and metastasis of colon cancer tumors.

Herein, we verified the inhibitory effect of Hesperidin on colon cancer cell proliferation, migration, and invasion by downregulating SLC5A1 and inhibiting EGFR phosphorylation. Simultaneously, SLC5A1 overexpression can significantly promote colon cancer cell proliferation, migration, and invasion and inhibit the anticancer effect of Hesperidin. This study may help identify Hesperidin as a new potential treatment for colon cancer.

## Materials and Methods

### Drugs

Hesperidin procured from MCE Shanghai, China (catalog number 520-26-3, with a purity of at least 98%) was dissolved in dimethyl sulfoxide to achieve a 10 mM concentration.

### Cell culture

The Cell Bank of the Chinese Academy of Sciences (Shanghai, China) provided the colon cancer cell lines HCT-116 and DLD-1 and the normal colonic epithelial cell line NCM460. DLD-1 cells were cultured in DMEM, while HCT-116 and NCM460 cells were incubated in RPMI 1640. Both media contained 10% fetal bovine serum (FBS) and were incubated at 37 °C with 5% CO_2_.

### Lentiviral transfection

Lentiviral vectors for knockdown (sh-SLC5A1) and overexpression (oe-SLC5A1) were obtained from Hanbio (Shanghai, China). The transfection procedures for all lentiviral vectors were performed according to the manufacturer's instructions. Table S1 presents the sequences of shRNA.

### Quantitative real-time polymerase chain reaction (qRT-PCR)

The Trizol method was used to extract total RNA, which was subsequently transcribed into cDNA (TransGen Biotech, Beijing, China). The cDNA was used for qRT-PCR analysis (TaKaRa, Biomedical Technology Co.). CT(2^-ΔΔCT^) was used for data analysis. Table S1 presents the primer sequences.

### Cell counting kit-8 (CCK8)

In the above-mentioned circumstances, 5,000 cells were placed in each of the 96-well plates and exposed to varying doses of Hesperidin. Cell viability was assessed after 48 h. Before the analysis, 20 µL of CCK-8 reagent was added to each well and incubated for 1.5 h. GraphPad Prism software (version 7.0; La Jolla, CA) was used to generate the proliferation curve based on an optical density of 450 nm. The experiment was repeated thrice.

### Colony formation assay

Approximately 1,000 cells were placed in a 6-well plate and exposed to different levels of Hesperidin for 48 h. The medium, supplemented with 10% FBS without Hesperidin, was refreshed for a continuous incubation period of 14 days. Afterward, the cells were fixed with 4% formaldehyde for 30 min and stained with 1% crystal violet for 6 h. Subsequently, ImageJ was used to visualize the assay.

### 5-Ethynyl-2'-Deoxyuridine (EdU) assay

The UE YF®594 Click-iT EdU kit was used for the Edu assay to assess cell proliferation. Cells were inoculated in 96-well plates (2×10^4^/well) for 24 h. After 48 h of Hesperidin treatment, fixation, permeabilities, and staining were performed following the instructions on the kit. A fluorescence microscope was used to observe the cells, and the rate of cell proliferation was measured.

### Wound-healing assay

Cells were inoculated in 6-well plates at 1×10^6^ cells/well and incubated at 37 °C to achieve 90% confluence. A 200 μL pipette tip was used to create a consistent scratch across the well. Afterward, the isolated cellular debris was removed using phosphate-buffered saline, and fresh media containing different concentrations of Hesperidin was added to the incubation. The progression of wound closure was observed at 0 and 48 h after the injury using an inverted microscope, with images captured at these intervals. The scratch area was quantified, and ImageJ software was used to calculate the rate of cell migration.

### Transwell assay

In the upper chamber, 200 µL of serum-free medium containing cells was added after 48 h of Hesperidin treatment, while 800 µL of a medium with 10% FBS was introduced into the lower chamber for culture. The porous membrane of the upper chamber was pre-treated with Matrigel matrix (Catalog #354230, BD, USA) to enhance cell invasion. After a 48-h incubation at 37 °C with 5% CO_2_, non-invading cells on the upper surface of the membrane were removed. The cells that migrated to the lower surface were fixed with a 4% formaldehyde solution, stained with crystal violet, and rinsed with tap water. ImageJ software was used to quantify the number of invasive cells.

### Western blotting

The cells were lysed using RIPA buffer. The BCA protein detection kit from Takara Biotechnology Co., Ltd. was utilized to quantify total protein levels. A 10% sodium dodecyl sulfate-polyacrylamide gel electrophoresis (SDS-PAGE) was used to separate 40 µg of the samples and subsequently transferred to a PVDF membrane (0.2μM, Merck Millipore, Darmstadt, Germany). The membranes were blocked by 5% skim milk for 120 min at room temperature and subsequently incubated overnight at 4 °C with one of the following primary antibodies: β-actin antibody (1:2,000; Serivicebio; GB11001), E-cadherin antibody (1:1,000; Affinity; AF0131), N-cadherin antibody (1:1,000; Affinity; AF5239), Vimentin antibody (1:1,000; Affinity; AF7013), SLC5A1 (1:1,000; Abcam; ab14686), Phospho-EGFRTyr1068 (1:10,00, Cell Signaling Technology; 3777, D7A5), EGFR (1:1,000; Cell Signaling Technology; 4267, D38B1), SLC5A1 (1:1,000; Origene; TA381679). After the primary antibody incubation, the membranes were exposed to the secondary antibody conjugated with HRP (1:8,000; Cell Signaling Technology). ChemiDocXRS + (Bio-Rad) was used to identify protein bands, and ImageJ software was used to analyze their intensities.

### Coimmunoprecipitation

Cells were lysed in a cell lysis buffer for Western blotting and immunoprecipitation, which contained protease inhibitors for 30 min at 4 °C. The specified antibodies and IgG agarose were included in the lysate and incubated overnight at 4 °C. The immunoprecipitates were washed thrice with a precooled 10% lysate solution to remove unbound proteins. The precipitates were resuspended in a 6×sodium dodecyl sulfate sample buffer. A 10% SDS-PAGE was used to isolate the separated proteins, which were analyzed using immunoblotting with the indicated antibodies.

### Statistical analysis

Each experiment was repeated thrice to guarantee its reliability. GraphPad Prism software (version 7.0) was used for data analysis. The data are presented as mean ± standard deviation. One-way analysis of variance was used to evaluate statistical variances between groups. A *p* < 0.05 was considered statistically significant.

## Results

### Hesperidin inhibits proliferation, migration and invasion of colon cancer cells

We cultured healthy human colon epithelial cells NCM460 and colon cancer cells HCT-116 and DLD-1 with different concentrations of Hesperidin (25, 50, 100, 200, and 400μM) for 48 h to examine the effect of hesperidin on colon cancer. The cell viability was measured using the CCK8 assay (Figure 1B). The results revealed that hesperidin, at 25 and 50μM, did not significantly affect cell viability. However, at 400μM, a significant reduction in viability was observed in the normal colonic epithelial cells NCM460. Consequently, we selected 100 and 200μM for subsequent analysis. We performed colony formation and EdU assays to evaluate the impact of hesperidin on colon cancer cell proliferation. After treatment with Hesperidin at 100 and 200μM, a significant reduction in colony formation was observed, indicating the inhibitory effect of Hesperidin on the proliferative capacity of these cells (Figure 1C). Similar outcomes were observed in the EdU assay (Figure 1D-E).

The wound healing assay revealed a significant decrease in the migration distance of colon cancer cells treated with Hesperidin at 100 and 200μM compared to those in the control group (Figure 1F-G). Consistent with the wound healing assay findings, the transwell assay revealed a significant decrease in the invasion rate of colon cancer cells after treatment with Hesperidin at 100 and 200μM (Figure 1H).

This study demonstrated that Hesperidin inhibits the invasive and migratory properties of colon cancer cells. Upon further examination, we observed alterations in the expression levels of epithelial-mesenchymal transition (EMT)-related proteins in HCT-116 and DLD-1 cells treated with Hesperidin at 100 and 200μM using Western blotting analysis. Notably, reduced levels of N-Cadherin, Vimentin, and Snail expression were observed, thus confirming the inhibitory role of Hesperidin in cell migration and invasion (Figure 1I).

### Hesperidin downregulates SLC5A1 in colon cancer cells

**Figure 2:**
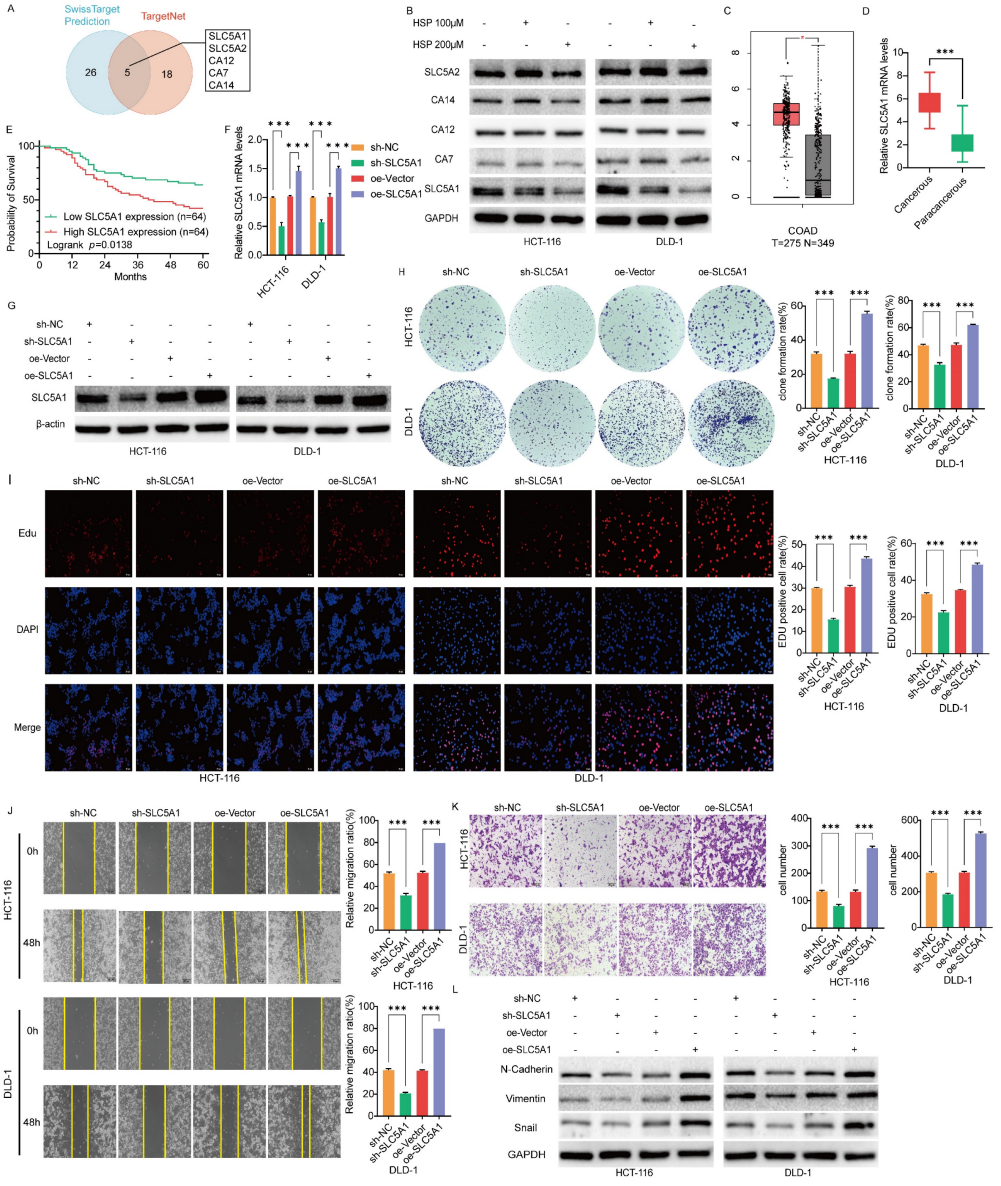
** SLC5A1 is downregulated by Hesperidin in colon cancer cells, is highly expressed in colon cancer cells, and is associated with poor prognosis of patients. SLC5A1 regulates the proliferation, migration and invasion of colon cancer cells. (A)** The Venn diagram shows five overlapping targets.** (B)** Protein expression levels of five targets in colon cancer cells under the action of 0, 100μM, and 200μM concentrations of Hesperidin. **(C)** The expression levels of SLC5A1 in human colon cancer and normal tissues were obtained from the GEPIA2.0 database. **(D)** The mRNA expression of SLC5A1 in our collected colon cancer samples (*n*=128; Student's t-test) **(E)** Kaplan-Meier curve of OS of SLC5A1 was performed using 128 colon cancer samples from the Second Affiliated Hospital of Nanchang University. **(F-G)** The lentiviral transfection efficiency was validated through qRT-PCR and Western blotting.** (H-I)** Colony formation assay and EdU assay were used to compare the proliferation ability of HCT-116 and DLD-1 cells in different groups.** (J)** Wound healing assays were performed to compare the migration ability of HCT-116 and LDL-1 in different groups** (K)** Transwell assays were performed to compare the invasion ability of HCT-116 and LDL-1 in different groups. **(L)** The expression of the biomarkers of EMT were detected by Western blotting. All experiments were repeated three times and the data were represented as mean±SD. *p<0.05; **p<0.01; ***p<0.001; ns, *p*>0.05.

Five potential binding targets for Hesperidin in colon cancer were identified to further investigate the potential mechanism of action in colon cancer alongside the predicted results from the SwissTarget Prediction and TargetNet databases (Figure 2A). SLC5A1 expression level was decreased in colon cancer cells treated with varying concentrations of Hesperidin compared to those in the control group (Figure 2B).

### SLC5A1 is significantly expressed in colon cancer cells and associated with poor prognosis in patients

The SLC5A1 expression profile obtained from the GEPIA 2.0 online database demonstrated that SLC5A1 is significantly expressed in colorectal cancer (Figure 2C). We also collected clinical samples from 128 pairs of colon cancer patients at the Second Affiliated Hospital of Nanchang University and performed qRT-PCR experiments, which showed that the mRNA levels of SLC5A1 were increased in all these colon cancer tissue samples as compared with the adjacent normal tissues (Figure 2D). The subsequent data was used to analyze the overall survival (OS) of the 128 patients. Based on the median expression level of cancerous tissue mRNA, 128 patients were divided into a low-expression group and a high-expression group for Kaplan-Meier survival analysis. The findings revealed that OS was significantly lower in the high-expression group than in the low-expression group, indicating a negative correlation between SLC5A1 expression level and prognosis of patients (*p* = 0.0138) (Figure 2E). Besides, we investigated the association between SLC5A1 mRNA expression levels and clinicopathologic features of patients with colon cancer. The findings revealed that elevated SLC5A1 expression was significantly correlated with tumor pathological stage (*p* = 0.013) and vascular invasion (*p* = 0.033) (Table 1).

### SLC5A1 inhibits proliferation, migration and invasion of colon cancer cells

Because of the high SLC5A1 expression in colon cancer cells, we inhibited endogenous SLC5A1 expression in HCT116 and DLD1 cells using the lentiviral SLC5A1 vector specifically targeting SLC5A1 (sh-SLC5A1). Moreover, we established two stable colon cancer cell lines that overexpress SLC5A1 (oe-SLC5A1). Figure 2F-G depicts their transfection efficiency. The transduction of oe-vectors or control viruses with shRNA (sh-NC) exhibited no effect on SLC5A1 expression. We subsequently tested colon cancer cell proliferation using EdU and colony formation assays. The findings revealed that after treatment with oe-SLC5A1, HCT-116 and DLD-1 cells significantly increased colony number and proliferative capacity. However, the knockdown of the HCT-116 and DLD-1 colon cancer cell lines (sh-SLC5A1) resulted in a decrease in the proliferative capacity of the cells (Figures 2H-I).

SLC5A1-mediated migration and invasion of colon cancer cells were further investigated. Wound healing and transwell assays revealed that oe-SLC5A1 significantly enhanced cell migration and invasion in both cancer cell types. However, SLC5A1 downregulation in HCT116 and DLD1 cells resulted in decreased cell migration and invasion (Figures 2J-K). These *in vitro* cell experiments indicate that elevated SLC5A1 levels are essential for colon cancer cell proliferation, migration, and invasion. Western blotting revealed that the expression of the biomarkers of EMT, N-Cadherin, Vimentin, and Snail was downregulated or upregulated after SLC5A1 knockdown or overexpression, which further demonstrated that SLC5A1 could regulate colon cancer cell migration and invasion (Figure 2L).

### SLC5A1 overexpression reverses the effect of Hesperidin on colon cancer cells

**Figure 3:**
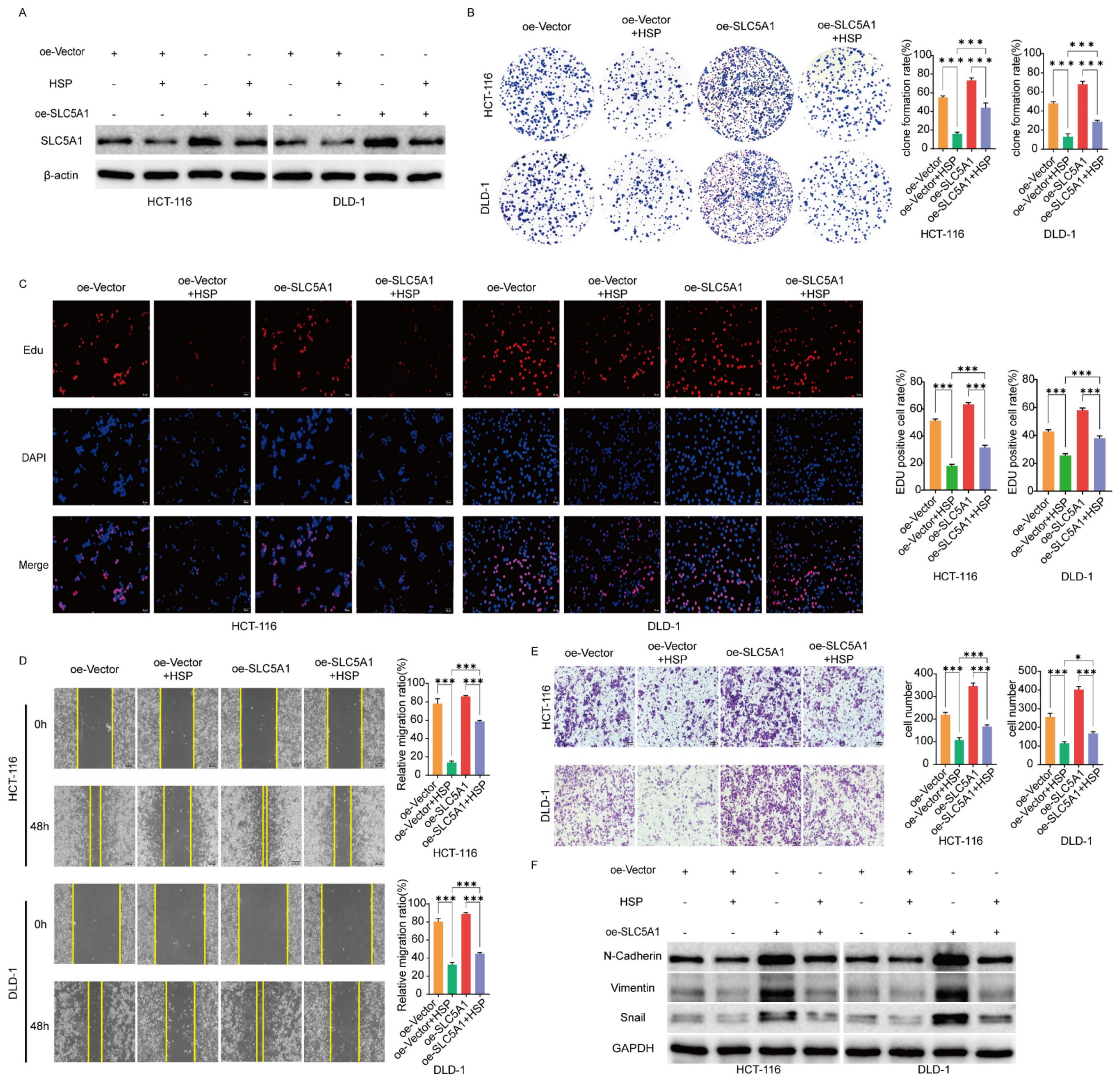
** SLC5A1 overexpression reverses the effect of Hesperidin on colon cancer cells. (A)** Control colon cancer cells and colon cancer cells overexpressing SLC5A1 by transfection with OE-SLC5A1 were exposed to 200μM Hesperidin for 48 h, and the expression of SLC5A1 was detected by Western blotting. **(B-C)** Colony formation assay and EdU assay showed that overexpression of SLC5A1 could eliminate the effect of Hesperidin on cell proliferation. **(D)** Wound healing assay showed that overexpression of SLC5A1 could eliminate the effect of Hesperidin on cell migration. **(E)** Transwell assay showed that overexpression of SLC5A1 could eliminate the effect of Hesperidin on cell invasion. **(F)** EMT-related proteins were detected by Western blotting. All experiments were repeated three times and the data were represented as mean±SD. *p<0.05; **p<0.01; ***p<0.001; ns, *p*>0.05.

Therefore, we hypothesized that Hesperidin, through SLC5A1, regulates the malignant progression of colon cancer. To validate the above hypotheses, we transfected HCT-116 and DLD-1 cells with oe-SLC5A1 to enhance their endogenous SLC5A1 expression and treated the transfected cells with HSP 200 μM. Western blotting revealed that the inhibitory effect of hesperidin on SLCA51 was partially mitigated by SLC5A1 overexpression in HCT-116 and DLD-1 cell lines (Figure 3A). We performed proliferation, migration, and invasion tests to better understand the effect of Hesperidin on the malignant progression of colorectal cancer through regulating SLC5A1. The results revealed that overexpression of SLC5A1 significantly enhanced the proliferation, migration, and invasion of colon cancer cells, an effect reversed upon the administration of Hesperidin to SLC5A1 overexpressing colon cancer cells (Figures 3B-E). The EMT-associated proteins (N-Cadherin, Vimentin, and Snail) exhibited similar patterns to those previously described (Figure 3F). The findings revealed that Hesperidin inhibits colon cancer proliferation, migration, and invasion by regulating SLC5A1.

### SLC5A1 regulates the proliferation, migration and invasion of colon cancer cells by regulating EGFR phosphorylation

**Figure 4:**
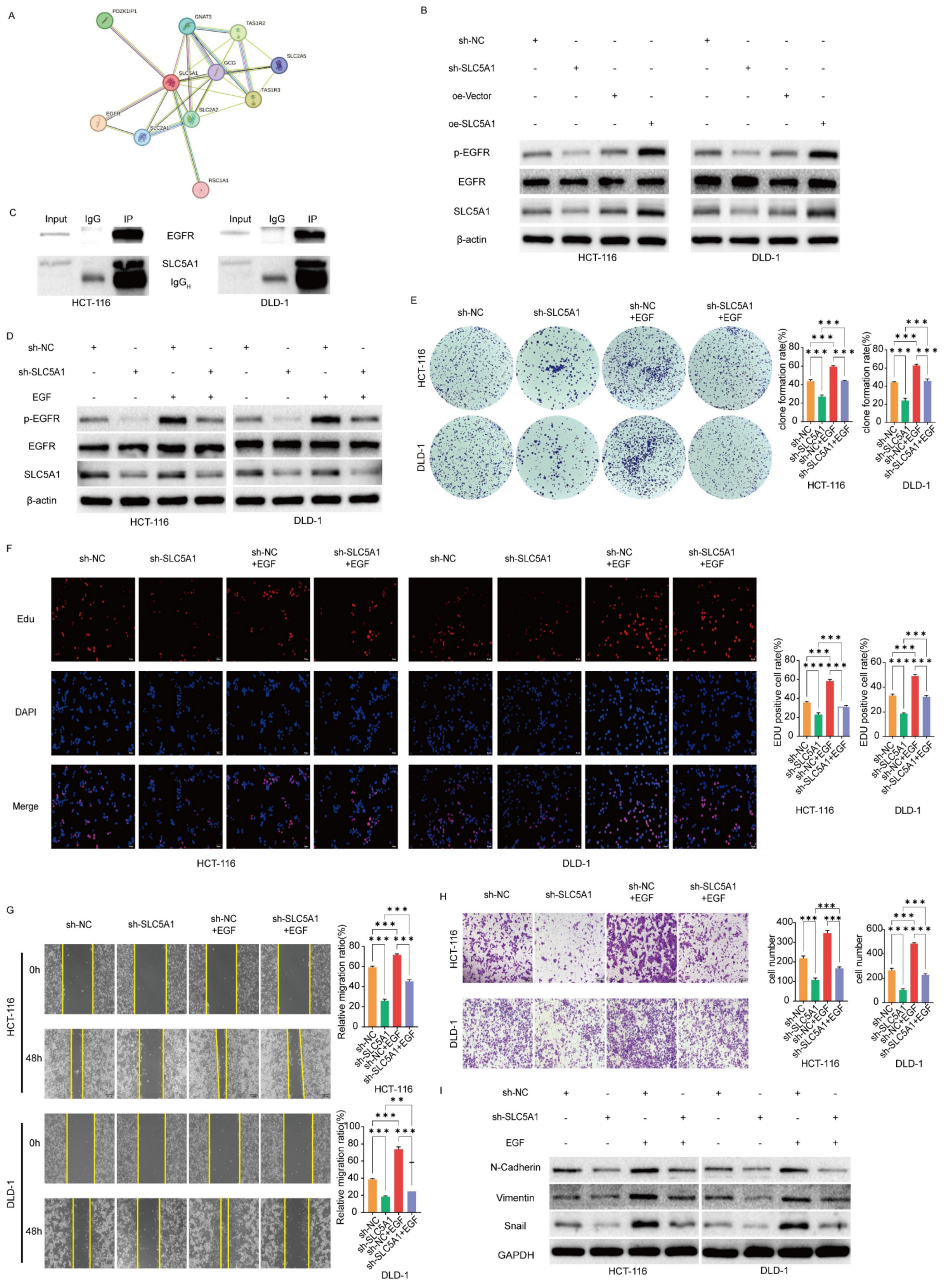
** SLC5A1 modulates the proliferation, migration and invasion of colon cancer cells by regulating EGFR phosphorylation. (A)** The protein-protein interaction network for SLC5A1. **(B)** Western blotting results showed that the expression of SLC5A1 was positively correlated with the phosphorylated EGFR protein.** (C)** Co-Immunoprecipitation was performed with indicated antibodies in HCT-116 and DLD-1 cells.** (D)** Control colon cancer cells and colon cancer cells knockdown SLC5A1 by transfection with sh-SLC5A1 were exposed to EGF for 48 h, the expression of SLC5A1 and phosphorylated EGFR were detected by Western blotting.** (E-F)** Colony formation assay and EdU assay showed that EGF could rescue the effect of SLC5A1 knockdown on cell proliferation.** (G)** Wound healing assay showed that EGF could rescue the effect of SLC5A1 knockdown on cell migration. **(H)** Transwell assay showed that EGF could rescue the effect of SLC5A1 knockdown on cell invasion. **(I)** The detection of EMT-associated proteins by Western blotting. All experiments were repeated three times and the data were represented as mean±SD. *p<0.05; **p<0.01; ***p<0.001.

To investigate how SLC5A1 influences colon cancer proliferation, migration, and invasion, we utilized the STRING database to predict potential proteins interacting with SLC5A1 (Figure 4A). Among the acquired proteins, EGFR is a protein associated with EMT 17-19. Consequently, we hypothesized that SLC5A1 may regulate the EGFR phosphorylation level, thereby influencing the proliferation, migration, and invasion of colon cancer. We performed preliminary verification through Western blotting experiments, which revealed that SLC5A1 knockdown or overexpression correspondingly decreased or increased EGFR phosphorylation level, while the protein level of EGFR remained constant across all conditions (Figure 4B). Furthermore, the coimmunoprecipitation assay corroborated the interaction between SLC5A1 and EGFR (Figure 4C).

### EGF reverses the inhibitory effect of SLC5A1 knockdown on the proliferation, migration and invasion of colon cancer cells

Upon administering EGF to SLC5A1 knockdown colon cancer cells, the EGFR phosphorylation level was restored (Figure 4D). We subsequently evaluated whether EGF could reverse the inhibitory effects of SLC5A1 downregulation on colon cancer cell proliferation, invasion, and migration. The colony formation, EdU, wound healing, and transwell assays were designed. These results indicate that EGF could reverse the proliferation, migration, and invasion abilities of colon cancer cells inhibited by SLC5A1 downregulation (Figures 4E-H). Furthermore, Western blotting revealed that EGF reversed the inhibitory effect of SLC5A1 knockdown on EMT (Figure 4I).

### Hesperidin downregulates the phosphorylation level of EGFR and EGF reverses this effect

**Figure 5:**
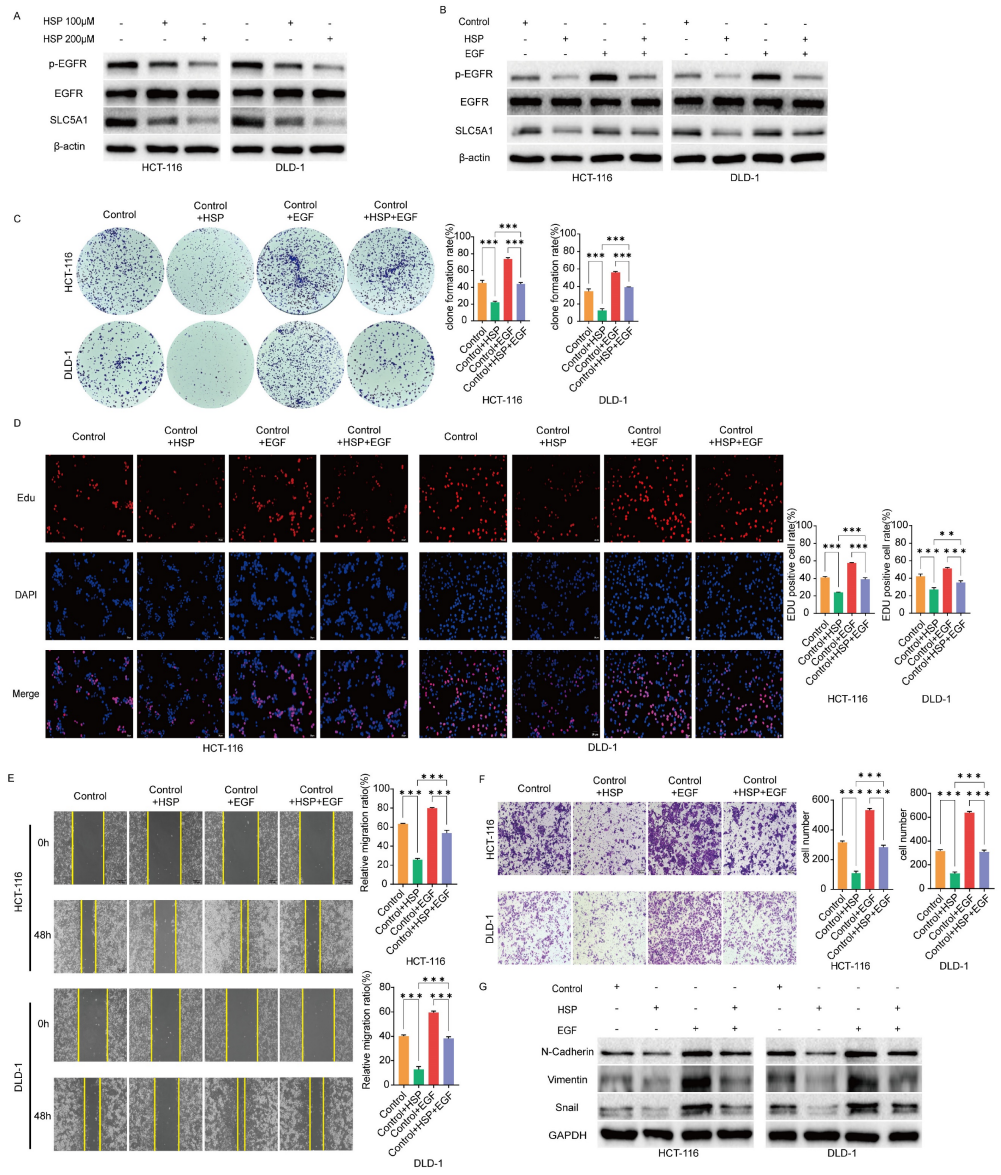
**Hesperidin downregulates the phosphorylation level of EGFR and EGF could reverse this effect. (A)** Western blotting results showed that Hesperidin could inhibit the phosphorylation of EGFR in colon cancer cells.** (B)** EGF reverses inhibition of EGFR phosphorylation by Hesperidin. **(C-D)** Colony formation assay and EdU assay showed the inhibitory effect of Hesperidin on the proliferation of colon cancer cells could be rescued by EGF. **(E)** Wound healing assay showed the inhibitory effect of Hesperidin on the migration of colon cancer cells could be rescued by EGF. **(F)** Transwell assay showed the inhibitory effect of Hesperidin on the invasion of colon cancer cells could be rescued by EGF. **(G)** Expression of EMT-related proteins in different groups assayed by western blotting. All experiments were repeated three times and the data were represented as mean±SD. *p<0.05; **p<0.01; ***p<0.001.

The above findings prompted us to investigate EGFR and its phosphorylated variants in colon cancer cells in untreated conditions and after treatment with Hesperidin at 100 and 200μM. The results revealed that EGFR phosphorylation levels were significantly reduced in the treated cells (Figure 5A). After adding hesperidin and EGF to colon cancer cells, the EGFR phosphorylation levels were more increased than those of colon cancer cells treated solely with Hesperidin, while the protein level of EGFR remained unchanged (Figure 5B). Similar outcomes and trends were observed in colony formation (Figure 5C), EdU (Figure 5D), wound healing (Figure 5E), transwell assay (Figure 5F), and expression of EMT-related proteins (Figure 5G). Based on these observations, we propose that Hesperidin exerts its anticancer effects in colon cancer cells by downregulating SLC5A1 expression, which diminishes EGFR phosphorylation.

## Discussion

Colon cancer is a common malignant tumor that significantly endangers human health. In recent decades, the prevalence of colon cancer has shifted toward younger populations and more advanced stages 20. Despite the rapid advancements in cancer screening techniques, a considerable number of individuals are still being diagnosed at a late stage with various symptoms, such as hematochezia or colonic obstruction 21, with approximately a quarter of patients already exhibiting signs of distant metastasis 22, 23. The treatment of colon cancer is still based on a surgical model supplemented by chemotherapy and radiotherapy, a model that creates a substantial physical, psychological, and financial burden on patients. Numerous studies reported that Hesperidin can inhibit various types of cancer. For example, it may hinder the progression of prostate cancer by inducing oxidative stress and disrupting Ca^2+^ balance 6. Moreover, it may inhibit the proliferation of breast cancer cells through the estrogen receptor/mitochondrial pathway 7. Furthermore, Hesperidin can induce apoptosis in HeLa cervical cancer by targeting Jab1 8. It can inhibit the advancement of lung cancer by targeting the miR-132/ZEB2 pathway 9. Lastly, it can inhibit the up-regulation of PD-L1 in oral cancer cells 24. This study demonstrated the anticancer properties of Hesperidin in colon cancer and, for the first time, investigated the potential molecular mechanisms underlying the invasive and metastatic inhibitory effects of Hesperidin in colon cancer. Our findings indicate that the ability of Hesperidin to prevent colon cancer may depend on the suppression of SLC5A1 expression, which inhibits EGFR phosphorylation. This study demonstrated that Hesperidin at < 400μM did not exhibit significant inhibitory effects on healthy human colon epithelial cells (NCM460) but could significantly inhibit the cell viability of colon cancer cells. The inhibitory effect of 400 μM Hesperidin on human normal colon epithelial cells NCM460 in the experiment may result from DMSO cytotoxicity, a solvent in Hesperidin solution. Subsequent functional experiments revealed that Hesperidin could impede colon cancer cell proliferation, migration, and invasion, implying that it can exert its anti-colon cancer effects *in vitro.*

SLC5A1, a constituent of the solute carrier family 5, commonly known as SGLT1, facilitates the cellular absorption of glucose. Several SLC carriers are not increased in cancer cells 14, 25 but have recently been recognized as markers of cancer characteristics 26, 27, especially SLC5A1, which is prevalent across various cancer types 28. Previous studies reported that SLC5A1 is associated with proliferation, migration, apoptosis, resistance to platinum-based chemotherapeutics, and poor prognosis in cancer cases 29-32. Consequently, medications that inhibit or reduce SLC5A1 activity may be effective in cancer treatment 33. SwissTarget Prediction and TargetNet forecast indicate that SLC5A1 is the only target with a binding probability exceeding 0.05, consistently identified in both databases, exhibiting expression changes in colon cancer cells treated with Hesperidin. Thus, we hypothesized that Hesperidin could inhibit cancer malignant progression by reducing SLC5A1 levels. Further research found that SLC5A1 is highly expressed in colon cancer. The high-expression group exhibited a more advanced pathological stage, a higher incidence of vascular invasion, and a shorter OS than the low-expression group. In addition, we observed that compared to the NC group, SLC5A1 knockdown could suppress the proliferation, migration, and invasion of colon cancer cells, while SLC5A1 overexpression could suppress the inhibitory effect of Hesperidin on colon cancer cells.

We utilized the STRING database to identify potential interacting proteins to clarify the molecular mechanism underlying the anticancer effects of hesperidin via SLC5A1. Among the acquired proteins, EGFR is a protein associated with EMT 17-19. EGFR phosphorylation facilitates the activation of downstream signaling molecules, including PI3K, AKT, and mTOR, and enhances colon cancer cell proliferation, migration, and invasion 34, 35. EMT is a well-documented process wherein epithelial tumor cells adopt a more migratory and aggressive phenotype, enhancing their invasive capacity 36. This transition confers stem cell-like characteristics to tumor cells and is implicated in cancer metastasis 18, 37. During EMT, the expression of epithelial markers, including E-cadherin, diminishes, while mesenchymal markers, including N-Cadherin and Vimentin, increase 37. Accordingly, we hypothesized that EGFR may be downstream of SLC5A1, affecting colon cancer cell migration and invasion. Western blotting revealed that overexpression or knockdown of SLC5A1 resulted in the same changes in p-EGFR levels, while EGFR levels remained unchanged. EGF addition mitigated the inhibitory effect of SLC5A1 knockdown. Our results demonstrated that Hesperidin reduced EGFR phosphorylation levels, a finding contrasts with observations made after EGF addition.

## Conclusion

This study demonstrated the anti-tumor effect of Hesperidin on colon cancer and provided preliminary evidence that hesperidin can impede the proliferation, migration, and invasion of colon cancer cells. Hesperidin mechanistically downregulated SLC5A1 expression, thereby inhibiting EGFR phosphorylation. Our findings may establish a theoretical and experimental basis for developing a new colon cancer drug.

## Supplementary Material

Supplementary information.

## Figures and Tables

**Table 1:** The relationship between SLC5A1 mRNA expression and clinicopathological characteristics

Variables	Clinicopathological characteristics	Numbers	SLC5A1 low expression	SLC5A1 high expression	*p* value
Age					
	≤60	59	28	31	0.595
	>60	69	36	33	
Gender					
	Female	59	29	30	0.859
	Male	69	35	34	
CEA					
	≤5	67	34	33	0.860
	>5	61	30	31	
TNM stage					
	I+II	56	35	21	**0.013**
	III+IV	72	29	43	
Vessel invasion					
	No	78	42	30	**0.033**
	Yes	34	22	34	
Tumor size(cm)					
	≤5	60	28	32	0.479
	>5	68	36	32	
