# Supplementary material for: Hesperidin inhibits colon cancer progression by downregulating SLC5A1 to suppress EGFR phosphorylation
Source: J Cancer. 2025 Jan 1;16(3):876–87. doi: 10.7150/jca.104867 (PMC11705064; doi:10.7150/jca.104867)
Supplement: Supplementary file 1 — Supplementary information. [file jcav16p0876s1.pdf]

The shRNA of SLC5A1(sh-SLC5A1) sequence: ATCTTTCTCTTATTGGCAA.

Forward Primer of SLC5A1: AGGCTATGACGCCTTCATGG.

Reverse Primer of SLC5A1: GGCCCTTGGAGTGTAGCATT.

Forward Primer of GAPDH: GAAGGTGAAGGTCGGAGTC.

Reverse Primer of GAPDH: GAAGATGGTGATGGGATTTC.
